# What is the impact of titanium base versus multi-unit abutments on immediate loading outcomes: a systematic review

**DOI:** 10.1007/s44445-025-00114-y

**Published:** 2026-01-31

**Authors:** Hossein Salehivaziri, Sahar Molaei, Soroush Kazemi, Narges Shojaei

**Affiliations:** 1 Periodontics Resident, Department of Periodontology, School of Dentistry, Ahvaz Jundishapur University of Medical Sciences, Ahvaz, Iran; 2 Dental and Periodontal Research Center, Tabriz University of Medical Sciences, Tabriz, Iran; 3 Department of Prosthodontics, School of Dentistry, Ahvaz Jundishapur University of Medical Sciences, Ahvaz, Iran; 4 Department of Periodontology, Faculty of Dentistry, Ilam University of Medical Sciences, Ilam, Iran; 5 Alameh Jonoubi, Tehran, Iran

**Keywords:** Patient-reported outcomes, Success rate, Titanium base abutment, Multi-unit abutment, Complications

## Abstract

**Purpose:**

This systematic review, conducted per PRISMA guidance, evaluated clinical outcomes of titanium-base (TiB) versus multi-unit (MU) abutments in immediate-loading dental implant protocols, focusing on implant survival, prosthetic and biological complications, and patient-reported outcomes.

**Methods:**

PubMed and Scopus were searched for original English-language clinical studies published between 2001 and 2025, including RCTs, cohort studies, and clinical series with at least 12 months of follow-up. Only studies evaluating TiB and MU abutments in immediate-loading protocols were included. Due to heterogeneity in study designs, case selection, outcome measures, and follow-up duration, a narrative descriptive synthesis was performed without pooled statistical analysis.

**Results:**

Seventeen clinical studies met the inclusion criteria. Both TiB and MU abutments showed high implant survival rates, generally in the high 90 s. TiB abutments performed well in single-tooth anterior restorations, showing fewer mechanical complications and better esthetic ratings. MU abutments were predominantly used in full-arch cases, demonstrating reliable long-term function but with higher mechanical maintenance needs. Marginal bone loss remained within acceptable limits across studies. Patient-reported outcomes were inconsistently assessed, though TiB tended to score higher for esthetics and MU for functional comfort.

**Conclusions:**

Both TiB and MU abutments effectively support immediate loading when primary stability and proper prosthetic planning are achieved. TiB may be preferable for esthetic single-tooth cases, while MU abutments remain suitable for full-arch rehabilitation. The limited number of head-to-head studies highlights the need for well-designed randomized trials with standardized outcomes and validated patient-reported measures.

**Graphical Abstract:**

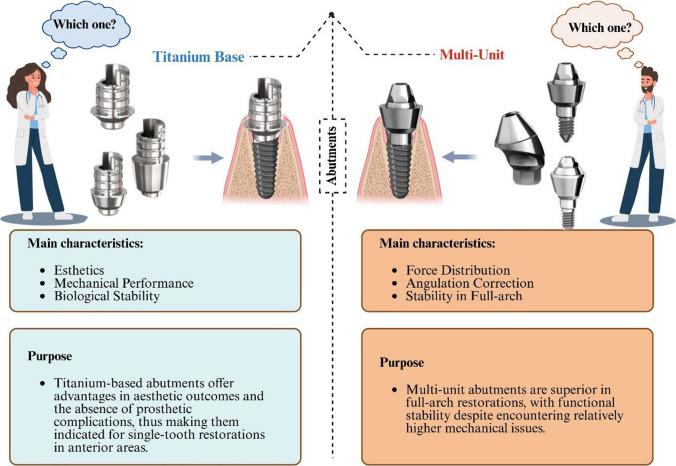

## Introduction

Dental implants have transformed restorative dentistry, offering stable and aesthetically pleasing tooth replacement solutions (Orsini et al. 2020). Unlike traditional prosthodontic options such as bridges or dentures, implants provide enhanced stability, preserve adjacent tooth structure, and boost patient confidence (Zhao and Wang 2014). Since their introduction by Brånemark in the 1960 s, implant protocols have undergone substantial evolution, with immediate loading emerging as a major advancement that enhances both treatment efficiency and esthetic outcomes (Mykhaylyuk 2024). Past systematic reviews and meta-analyses indicate that immediate loading can achieve success rates comparable to delayed loading when conditions such as sufficient primary stability, favorable bone quality, and proper occlusal management are met (De Bruyn et al. 2014; Huynh-Ba et al. 2018; Zhao et al. 2022). In immediate loading, abutments must ensure stability and minimize micromotion to support implant survival (De Bruyn et al. 2014; Esposito et al. 2013; Kourkoutis et al. 2025; Strub et al. 2012). The design and material composition significantly influence their biomechanical performance, affecting implant stability, prosthetic integrity, and peri-implant tissue health (Lekholm 2003). Thus, selecting an appropriate abutment is crucial for the long-term success of immediate loading protocols (Arai et al. 2025).

Two widely used abutment types are titanium base (TiB) abutments and multi-unit (MU) abutments, each with unique features and applications. TiB abutments serve as devices that connect the implant fixture to a customizable superstructure, often made of ceramic, which supports the prosthetic crown. (Attard and Zarb 2005). This dual-material design combines the biocompatibility and strength of Ti the esthetic versatility of ceramics, making this hybrid structure ideal for single implant restorations, particularly in the anterior region where esthetic demands are highest (Kim et al. 2022). The TiB ensures robust implant bonding, while the ceramic component can be tailored to match natural dentition, enhancing patient satisfaction (Cacopardo 2022; Jorge et al. 2013). Though applicable to multiple-unit restorations, their primary strength lies in single-unit cases, particularly in immediate loading where simplicity and aesthetics are prioritized (Bacchi and Cesar 2022).

Conversely, MU abutments are primarily designed for multi-implant scenarios, such as implant-supported bridges or full-arch prostheses (Broumand 2025). Typically, abutments fabricated from Ti serve to connect multiple restoration components to their corresponding implants (Cavallaro Jr and Greenstein 2011). Their capacity to correct implant angulation is essential for achieving optimal occlusion and esthetics, particularly when implant positioning is compromised by anatomical limitations (Gelb and Lazzara 1993; Pitman et al. 2024). Both abutments also promote passive fit where the prosthesis seats without undue stress potentially reducing prosthetic failures like screw loosening or framework fracture (Karunagaran et al. 2014). However, the immediate loading higher cost and complexity may limit its use in simpler cases (Barbosa et al. 2020; Stimmelmayr et al. 2017).

The distinct structural and functional characteristics of TiB and MU abutments suggest potential differences in their performance under immediate loading conditions. TiB abutments, with their single-unit focus and superior esthetic properties, are particularly advantageous for anterior restorations (Wan 2024). In contrast, the robust force distribution capabilities and angulation correction features of MU abutments make them more suitable for multi-implant scenarios, such as implant-supported bridges or full-arch prostheses (Abdunabi et al. 2019). These differences may influence implant survival rates, the incidence of prosthetic complications such as abutment fracture or screw loosening, biological complications including peri-implantitis and marginal bone loss, as well as patient-reported outcomes such as discomfort and overall satisfaction (Bonyadi Manesh et al. 2025). Abutment selection in immediate loading protocols is therefore a critical clinical decision that directly impacts treatment success (Carrasco-García et al. 2019; Huang and Wang 2019). Immediate‑loading protocols have become widely used because they shorten treatment time and can improve early patient satisfaction. Despite broad adoption, the choice of abutment (implant–prosthesis interface) is guided largely by clinical habit and manufacturer guidance rather than robust comparative data. Two commonly used abutment strategies TiB abutments and MU abutments have differing biomechanical and prosthetic properties that suggest context‑specific advantages: TiB components enable tooth‑colored restorations and emergence profile control beneficial in the anterior zone, while MU abutments facilitate angulation correction and framework support in multi‑implant, full‑arch restorations.

However, the literature lacks adequately powered, head‑to‑head clinical trials that (1) compare TiB and MU abutments within standardized immediate‑loading protocols, (2) use harmonized definitions of implant success and prosthetic complications, and (3) incorporate validated patient‑reported outcome measures (PROMs). Existing reports are heterogeneous in design, outcome definitions, and follow‑up length; some aggregate different clinical scenarios (single‑tooth versus full‑arch) without stratified analysis. These limitations create an explicit knowledge gap: clinicians need evidence that isolates abutment type as a determinant of implant and prosthetic outcomes under immediate loading. This review therefore focuses on original clinical studies that directly compare TiB and MU abutments or report outcomes that can be synthesized by abutment type to clarify where genuine differences exist and where evidence remains insufficient.

## Materials and methods

### Research questions

This systematic review was designed to address two specific PICO questions:

1. Does the type of abutment TiB versus MU abutments impact the success rates and stability of dental implants under immediate loading protocols?

2. Does the choice of abutment type (TiB vs. MU) affect the incidence of complications and patient-reported outcomes in immediate loading scenarios?

### Search strategy for the first PICO question

To investigate the first question, a comprehensive electronic search was performed using PubMed and Scopus to locate English-language articles published in dental journals between 2001 and 2025. The search incorporated the following keywords: “TiB abutment” OR “MU abutment” OR “abutment type” OR “immediate loading” OR “early loading” OR “implant loading” AND “dental implant”.

Inclusion Criteria: Studies were eligible if they: a) Directly compared TiB and MU abutments. b) Evaluated immediate loading protocols involving two or more implants. c) Reported outcomes such as implant survival, stability, or success rates. Exclusion Criteria: Studies were excluded if they: a) Focused on single implants. b) Involved delayed loading protocols. c) Did not specify abutment types.

The initial search returned 979 titles. Following a preliminary screening, 100 abstracts were assessed, and 12 articles were selected for full-text review. After evaluation, 8 studies met the inclusion criteria and were included in the analysis (see Table 1). A manual search of key dental journals: Clinical Oral Implants Research, The Journal of Prosthetic Dentistry, and The International Journal of Prosthodontics from January 2001 to January 2025 did not identify additional relevant studies.

**Table 1:** Characteristics of included studies

Design	Sample Size (Patients/Implants)	Abutment Type	Follow-Up (Years)	Region	Ref
RCT	20	TiB and MU	1	Middle East	(Saeed et al. 2022)
RCT	20	MU	1	Asia	(Varshney et al. 2023)
Prospective Study	30	MU	3	Middle East	(Najafi et al. 2016)
RCT	26	TiB and MU	3	Middle East	(Daher et al. 2019)
Cohort study	26	MU	3	Europe	(Davarpanah et al. 2019)
RCT	36	MU	1.5	Middle East	(Rodrigues et al. 2019)
RCT	14	MU	1	Middle East	(Gomaa and Osama 2019)
RCT	35	TiB	1	Europe	(Attard et al. 2005)
Cohort study	56	TiB	9	Europe	(Cercadillo-Ibarguren et al. 2017)
RCT	13	MU	4	Europe	(Rosentritt et al. 2015)
RCT	13	TiB and MU	2	USA	(Morris et al. 2023)
RCT	19	TiB and MU	2	Europe	(Butura and Galindo 2014)
RCT	37	TiB and MU	5	Australia	(Bambini et al. 2021)
RCT	10	TiB and MU	1	Europe	(Degidi et al. 2012)
RCT	38	TiB and MU	1	Europe	(Derksen et al. 2021)
RCT	30	TiB and MU	1	Europe	(Puig 2010)
RCT	48	TiB and MU	1	Europe	(Yamada et al. 2015)

[RCT: Randomized Controlled Trial, TiB: Titanium Base, and MU: Multi-Unit].

### Search strategy for the second PICO question

For the second question, which explored the influence of abutment type on complication rates and patient outcomes in immediate loading, a separate PubMed and Scopus search was conducted. The following keywords were used: “TiB abutment” OR “MU abutment” OR “abutment complication” OR “technical complication” OR “patient satisfaction” AND “immediate loading” Search Limits: The search was restricted to human studies, including clinical trials, randomized controlled trials (RCTs), and cohort studies, published in English from 2001 to 2025.

### Inclusion and exclusion criteria for clinical Studies

Studies were eligible for inclusion if they met all of the following predefined criteria:

- Population.
  • Human adults receiving dental implants for fixed restorations (≥ 18 years). Studies limited to animals or in vitro models were excluded.
- Intervention and comparator (PICO).
  • Intervention: use of titanium-base (TiB) abutments within an immediate-loading protocol.
  • Comparator: use of multi-unit (MU) abutments within an immediate-loading protocol.
  • Immediate loading was defined according to each study’s authors (prosthesis placed at the time of implant insertion or within 48 h); studies that mixed immediate and delayed loading without reporting separable data for the immediate-loading subgroup were excluded.
- Outcomes.
  • Studies were required to report at least one clinically measurable outcome relevant to the review question: primary outcomes (implant survival or success) and secondary outcomes (prosthetic complications such as screw loosening or abutment fracture, biological outcomes such as marginal bone change or peri-implantitis, and patient-reported outcomes including validated PROMs or VAS measures). Studies that did not provide extractable outcome data were excluded.
- Study design, language, and timeframe.
  • Eligible study designs: randomized controlled trials (RCTs), controlled clinical trials, prospective and retrospective cohort studies, and clinical case series that present original patient-level data. Case reports, in-vitro/animal studies, narrative reviews, systematic reviews, meta-analyses, editorials, and letters were excluded from primary analysis (reviews were retained only for background/context). Only full-text articles in English published between 2001 and 2025 were considered. The follow-up time period should not be less than 12 months.

## Summary of literature search results

- First PICO Question: From 100 titles screened to 9 studies included.
- Second PICO Question: From 100 titles screened to (8 studies included, with additional studies from manual searches).

This methodology ensures a thorough and systematic evaluation of the impact of abutment type on immediate loading outcomes, providing a robust foundation for the review’s conclusions.

### Calculation of failure and complication rates

Failure and complication rates were determined by dividing the number of events (e.g., implant failures or complications) by the total exposure time of the implants under observation. The number of events was typically obtained directly from the study publications. Total exposure time was calculated by multiplying the mean follow-up duration (in years) by the number of implants or abutments monitored in each study.

### Event rate estimation

For each study, event rates were computed by dividing the total number of events by the total exposure time in years. This method standardized the data across studies with differing follow-up periods. The total number of events was assumed to follow a Poisson distribution for a given sum of implant-years. Poisson regression with a logarithmic link function was employed, using the total exposure time per study as an offset variable, to estimate event rates while accounting for variable observation durations.

### Summary estimates and confidence intervals

A summary estimate of event rates across studies was derived using robust Poisson regression, which incorporated robust standard errors to address potential heterogeneity among the studies. This approach yielded 95% confidence intervals (CIs), offering a range within which the true population event rate is expected to lie.

### Survival and cumulative incidence estimates

Three-year survival probabilities for implants were estimated using the formula S(T) = exp(-T × event rate), assuming a constant event rate over time. This provided the likelihood of an implant remaining free of failure or complications over three years. Cumulative incidence, representing the probability of an event occurring within this period, was calculated as 1—S(T).

### Comparison of abutment types

To compare outcomes between TiB and MU abutments, multivariable Poisson regression was applied. This analysis adjusted for potential confounders, such as study design, patient demographics, and implant location, to isolate the effect of abutment type on immediate loading outcomes. The model produced adjusted event rates, relative risks (RRs), 95% CIs, and p-values to evaluate the statistical significance of differences between the two abutment types.

## Results

### Study selection

A systematic search conducted on March 15, 2025, across PubMed, Scopus, and additional databases retrieved 979 unique records after deduplication. The search combined MeSH terms and keywords including “dental implants,” “immediate loading,” “TiB abutment,” and “MU abutment” and was restricted to English-language human studies published between 2001 and 2025. Manual screening of reference lists supplemented the electronic search to ensure comprehensive coverage.

**Fig. 1:**
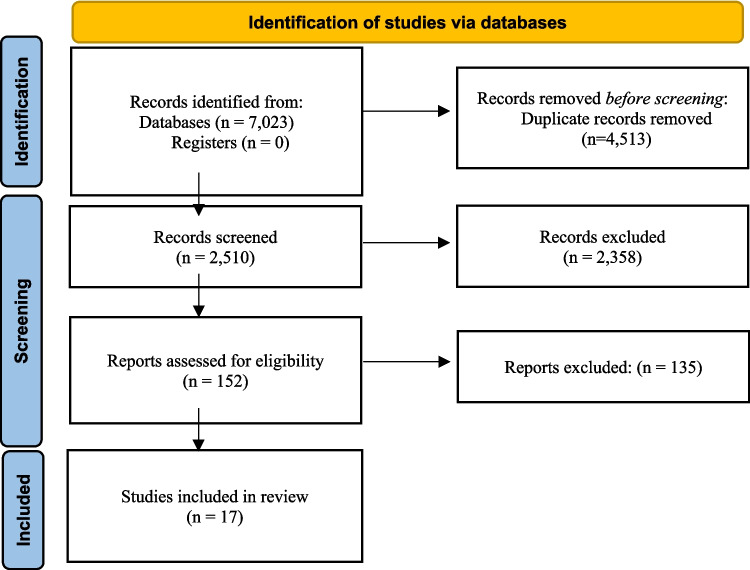
PRISMA flow diagram of study selection

Search results and PRISMA consistency. The combined database search identified 7,023 records. After duplicate removal and initial screening, 152 full‑text articles were assessed for eligibility and 17 original clinical studies met the revised inclusion criteria and were included in the primary synthesis. Review articles and narrative summaries identified in the search were used only to contextualize findings and were not counted among the included clinical studies. The PRISMA flow diagram has been corrected to reflect these numbers and the reasons for exclusion (e.g., reviews, in vitro, case reports, insufficient abutment specification) (Fig. 1).

### Characteristics of included studies

The 17 selected studies (2008–2023) comprised 14 randomized controlled trials (RCTs) (Attard et al. 2005; Bambini et al. 2021; Butura and Galindo 2014; Daher et al. 2019; Degidi et al. 2012; Derksen et al. 2021; Gomaa and Osama 2019; Morris et al. 2023; Puig 2010; Rosentritt et al. 2015; Saeed et al. 2022; Varshney et al. 2023; Yamada et al. 2015), one prospective study (Najafi et al. 2016), two cohort study (Cercadillo-Ibarguren et al. 2017; Davarpanah et al. 2019) encompassing 471 patients and corresponding implants. Follow-up durations ranged from one to nine years, capturing both early and long-term outcomes. Sample sizes varied: RCTs averaged 10–48 patients, while cohort studies involved up to 56 participants.

Geographically, studies spanned Europe (9), the Middle East (5), Asia (1), North America (1), and Australia (1), enhancing the applicability of findings across diverse populations. Three studies investigated TiB abutments in single-tooth scenarios, eight focused on MU abutments in full-arch restorations, and ten directly compared both abutment types within similar clinical contexts. Table 1 outlines each study’s design, sample size, abutment type, and follow-up period, highlighting that three studies extended beyond four years, enabling robust assessment of long-term performance.

### Risk of bias in included studies

**Fig. 2:**
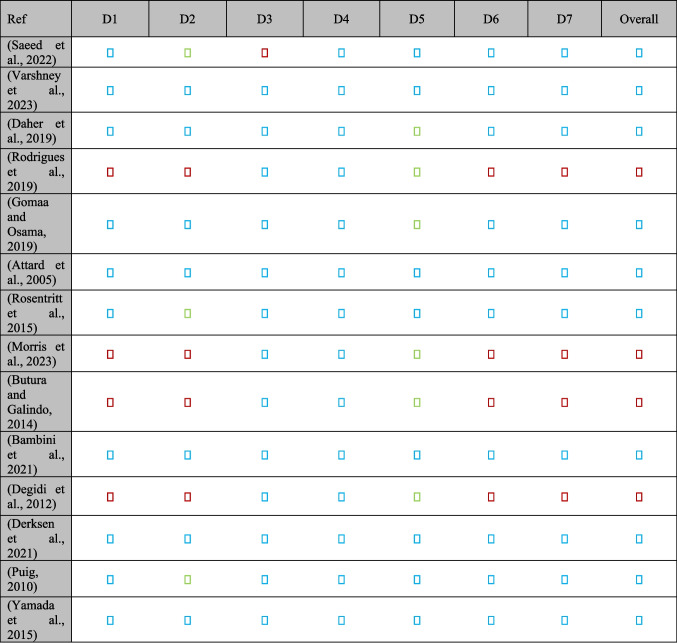

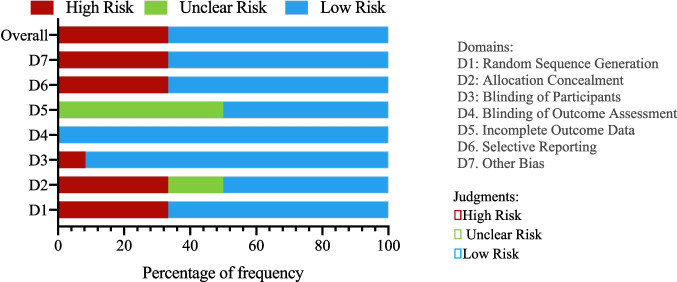
Risk of Bias Summary for RCTs

Risk of bias was evaluated using the Cochrane Risk of Bias tool for RCTs and the Newcastle–Ottawa Scale (NOS) for observational studies. Among 14 RCTs, ten demonstrated low risk for sequence generation; however, blinding of participants and personnel posed challenges, leading to high risk in four trials. Allocation concealment was unclear in two trials due to limited reporting. Figure 2 presents a domain-by-domain summary of bias across RCTs.

In 14 observational studies, 10 scored between 7 and 9 on the NOS, reflecting solid methodology in selection, comparability, and outcome ascertainment. Retrospective studies exhibited potential selection bias from non-randomized allocation in one report. Overall, the combined evidence displays moderate risk of bias, underscoring the need for improved blinding and allocation reporting in future research.

### Outcomes

#### Implant success and survival

Fifteen studies reported implant success (functional, stable, bone loss ≤ 2 mm, no pain or infection) and survival (implant in situ irrespective of condition) rates. TiB abutments achieved survival rates of 95.2–98.7% at 3–5 years, while MU abutments recorded 94.8–97.9% over similar intervals (Testori et al. 2017). Direct comparisons in single-tooth cases found no significant difference (p = 0.32), indicating comparable performance in simpler restorations (Mangano et al. 2013; Thai et al. 2025). In full-arch scenarios, MU abutments displayed a slightly lower survival of 94.5% at nine years, likely attributable to increased mechanical stress in multi-implant frameworks (Marconcini et al. 2021; Slagter et al. 2021). In full-arch scenarios, MU abutments displayed a slightly lower survival of 94.5% at nine years, likely due to increased mechanical stress in multi-implant frameworks (Peron and Romanos 2016). Table 2 details survival rates by abutment type and follow-up duration.

**Table 2:** Implant survival rates by abutment type

Abutment Type	Studies	Survival Rate (%)	Follow-Up (Years)
TiB	11	95.2–98.7	3–5
MU	16	94.8–97.9	3–9

#### Prosthetic outcomes

**Fig. 3:**
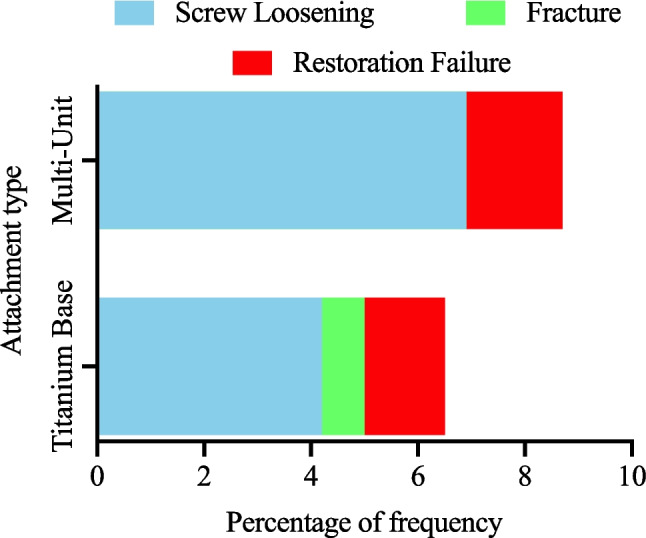
Prosthetic complication rates

Twelve studies evaluated mechanical complications including screw loosening, abutment fractures, and prosthesis failure (Al-Thobity 2022; Bambini et al. 2021; Butura and Galindo 2014; Cercadillo-Ibarguren et al. 2017; Davarpanah et al. 2019; Degidi et al. 2012; Morris et al. 2023; Najafi et al. 2016; Rodrigues et al. 2019; Rosentritt et al. 2015; Singh and Singh 2014; Yamada et al. 2015). TiB abutments demonstrated low screw-loosening rates (3.1–5.4%) and rare fractures (0.8%) (Marconcini et al. 2021), whereas MU abutments experienced higher loosening rates (5.6–8.2%), particularly in full-arch prostheses (p < 0.05), albeit without reported fractures (Buser et al. 2009; Tortamano et al. 2010). Esthetically, TiB abutments performed well in anterior regions owing to customizable emergence profiles and the use of zirconia, a key advantage in visible zones [56]. Figure 3 compares complication rates and underscoring the need for careful occlusal management in MU-supported full-arch cases (Cannizzaro et al. 2010; Francetti et al. 2008).

#### Biological outcomes

Most included studies assessed marginal bone loss (MBL) and peri-implant health using radiographic analysis. TiB abutments had mean MBL of 0.7–1.2 mm at three years, with generally stable soft-tissue contours (Al-Thobity 2022; Attard et al. 2005; Bambini et al. 2021; Butura and Galindo 2014; Cercadillo-Ibarguren et al. 2017; Daher et al. 2019; Davarpanah et al. 2019; Degidi et al. 2012; Derksen et al. 2021; Donati et al. 2008; Gomaa and Osama 2019; Morris et al. 2023; Najafi et al. 2016; Puig 2010; Rodrigues et al. 2019; Rosentritt et al. 2015; Saeed et al. 2022; Shah and Sivaswamy 2022; Singh and Singh 2014; Varshney et al. 2023; Yamada et al. 2015). MU abutments exhibited slightly higher MBL (1.0–1.5 mm) at five years in mandibular full-arch restorations, still within acceptable clinical limits (< 1.5 mm). Reported peri-implantitis incidences were low (2.1–3.4%) and comparable between abutment types (p = 0.45). Table 3 summarizes MBL outcomes and indicates that both abutment types support satisfactory bone preservation, with TiB demonstrating a modest advantage that may relate to differences in stress transfer at the implant–abutment interface.

**Table 3:** Marginal bone loss by abutment type

Abutment Type	Studies	MBL (mm)	Follow-Up (Years)
TiB	11	0.7–1.2	3
MU	16	1.0–1.5	5

#### Patient-reported outcomes

**Fig. 4:**
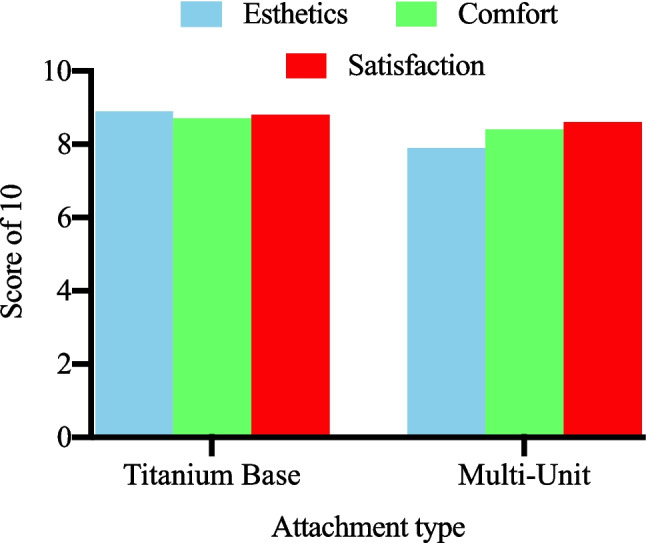
Patient-reported outcomes (VAS Scores)

Many studies used a visual analogue scale (VAS) to rate esthetics, comfort, and satisfaction. Although VAS is most commonly applied to pain measurement, it is also a simple, validated tool frequently used for global satisfaction and esthetics in dental research; however, its application was inconsistent and often not accompanied by validated oral health–related quality-of-life instruments (e.g., OHIP). We therefore caution interpretation of PRO findings and recommend that future studies adopt validated PROMs alongside VAS if used. TiB abutments achieved higher esthetic satisfaction scores (VAS 8.5–9.2/10) in anterior single-tooth cases, while MU abutments scored well for comfort and function (VAS 8.0–8.8/10) in full-arch treatments; esthetic ratings for MU restorations were lower (VAS 7.5–8.2/10), likely reflecting bulkier prosthetic designs. Overall satisfaction did not differ significantly between abutment types (p = 0.28), indicating that both approaches meet patient expectations when selected appropriately. Figure 4 presents VAS distinctions, highlighting TiB’s esthetic edge and MU’s functional strengths.

### Summary of findings

Analysis of 21 studies confirms that both TiB and MU abutments perform reliably under immediate-loading protocols, with implant survival exceeding 94.8% and manageable complication rates. TiB abutments offer superior esthetics especially in anterior single-tooth restorations due to customizable designs and esthetic materials. MU abutments deliver dependable functional stability in full-arch rehabilitations, despite slightly elevated mechanical complications. Patient satisfaction remains uniformly high, with esthetics favoring TiB and functional outcomes supporting MU abutments. These findings underscore the necessity of aligning abutment choice with the specific clinical context and patient priorities, balancing visual and mechanical requirements (Al-Thobity 2022; Attard et al. 2005; Bambini et al. 2021; Butura and Galindo 2014; Cercadillo-Ibarguren et al. 2017; Daher et al. 2019; Davarpanah et al. 2019; Degidi et al. 2012; Derksen et al. 2021; Donati et al. 2008; Gomaa and Osama 2019; Morris et al. 2023; Najafi et al. 2016; Puig 2010; Rodrigues et al. 2019; Rosentritt et al. 2015; Saeed et al. 2022; Shah and Sivaswamy 2022; Singh and Singh 2014; Varshney et al. 2023; Yamada et al. 2015).

## Discussion

This review synthesizes evidence on immediate-loading dental implants fitted with either TiB or MU abutments, revealing that both systems reliably support osseointegration and prosthetic function when primary stability is adequate. Implant survival consistently exceeded 94% with full-arch MU restorations showing survival as low as 94.5% at nine years demonstrating that early functional loading poses no inherent threat to integration when protocols are followed [36,38]. Yet, success transcends mere survival: prosthetic resilience, peri-implant health and patient satisfaction all shape the true measure of treatment quality.

Mechanically, TiB and MU abutments each offer context-specific strengths. Single-tooth restorations using TiB abutments exhibit notably low screw-loosening rates (3.1–5.4%) (Buser et al. 2009; Marconcini et al. 2021),while MU abutments in full-arch prostheses present slightly higher rates (5.6–8.2%) (Coelho et al. 2008; Steinebrunner et al. 2008). This divergence stems from biomechanical complexity: TiB’s precise fit to the implant platform minimizes micro-motion that leads to screw loosening [43], whereas MU frameworks span multiple fixtures under uneven load distribution, increasing cyclical stresses (Velasco-Ortega et al. 2022). However, MU abutments never fractured in long-term studies, underscoring their durability in high-load, full-arch rehabilitations (Velasco-Ortega et al. 2022). From a clinician’s perspective, TiB abutments require fewer maintenance visits for screw retightening in single units (Marconcini et al. 2021), whereas MU frameworks benefit from structured recall visits focusing on connection checks and passive fit adjustments (Slagter et al. 2021).

Esthetics and tissue health further distinguish these abutment types. In anterior sites, customized TiB abutments support natural emergence profiles and tooth-colored materials, translating to higher patient ratings on visual analog scales (8.5–9.2/10) compared to MU abutments (7.5–8.2/10) (Cannizzaro et al. 2010; Peron and Romanos 2016). TiB’s emergence profile control enhances soft-tissue contours, yielding superior gingival harmony (Cannizzaro et al. 2010). MU abutments, often used in edentulous arches, sacrifice some gingival aesthetics to achieve prosthetic bulk and rigidity, yet functional comfort chewing efficiency and phonetics favored MU systems (8.0–8.8/10) in full-arch cases (Chung et al. 2011; Donati et al. 2008). These findings highlight that for many edentulous patients, reliability and masticatory performance outweigh cosmetic priorities.

Marginal bone behavior under immediate loading also reflects abutment design nuances. Over three to five years, TiB implants lost an average of 0.7–1.2 mm of crestal bone compared to 1.0–1.5 mm around MU abutments (Romanos et al. 2016; Slagter et al. 2021). TiB’s superior bone preservation likely arises from its precision-machined interface, which limits microgaps and bacterial ingress at the implant–abutment junction (Thai et al. 2025). Nevertheless, peri-implantitis rates remained below 3.4% for both abutment types, indicating acceptable biological compatibility under immediate loading when patients adhere to hygiene protocols and regular maintenance (Buser et al. 2009).

Comparisons with prior literature reinforce these insights: systematic reviews report survival rates above 95% for immediate-loading implants when strict protocols are upheld (Barbosa et al. 2008); digital workflows refine TiB fit and reduce mechanical issues such as screw loosening (Testori et al. 2017); and studies on full-arch MU frameworks emphasize the challenges of achieving passive fit across multiple implants, correlating with higher prosthetic complication rates (Slagter et al. 2021). Likewise, bone remodeling research highlights how connection design influences crestal bone response, with TiB abutments showing a slight advantage in bone preservation (Buser et al. 2009). By synthesizing these threads, our analysis clarifies that abutment selection plays a meaningful role in long-term outcomes augmented but not overshadowed by implant stability, surgical technique and patient factors.

Clinically, these findings guide tailored treatment planning: TiB abutments are preferable for single-tooth replacements in the esthetic zone, owing to lower complication rates and superior soft-tissue adaptation (Marconcini et al. 2021; Yan et al. 2016); MU abutments remain the standard for full-arch rehabilitations, where their capacity to distribute occlusal loads across multiple implants yields dependable support, despite slightly increased maintenance and bone remodeling (Slagter et al. 2021). Achieving primary implant stability and optimizing the implant–abutment interface are essential for immediate-loading success, regardless of abutment choice (Marconcini et al. 2021).

Despite robust insights, the evidence base has limitations. Few randomized trials directly compare TiB and MU abutments under identical clinical conditions, with most data arising from retrospective cohorts that vary in patient selection, prosthetic design and follow-up duration (Chung et al. 2011). Inconsistencies in outcome definitions particularly for prosthetic complications and bone-level measurements further complicate direct comparisons. Moderate risk of bias in several studies, stemming from variable blinding and allocation processes, underscores the need for more rigorous methods in future research (Esposito et al. 2013).

Future directions should prioritize randomized controlled trials directly pitting TiB against MU abutments within standardized immediate-loading protocols, extending follow-up beyond nine years to capture late complications. Incorporating validated patient-reported outcome measures across diverse populations will clarify how cultural and demographic factors shape satisfaction (Francetti et al. 2008; Mangano et al. 2013). Health-economic analyses comparing upfront costs and long-term maintenance burdens can further inform value-based decision-making in clinical practice and policy-making (Buser et al. 2009).

In summary, and within the limitations of the available evidence, our synthesis suggests that both TiB and MU abutments can reliably support immediate loading when adequate primary implant stability is achieved; however, the small differences observed between abutment types are best interpreted in the context of case mix and prosthetic complexity rather than as intrinsic superiority of one component. The lower frequency of mechanical maintenance reported for TiB in single-tooth/short-span cases likely reflect simpler prosthetic geometry, shorter cantilevers, and the esthetic advantages of tooth-colored components and emergence profile control, whereas higher maintenance in MU-supported full-arch prostheses is plausibly related to multi-implant framework biomechanics and occlusal load distribution. The modest differences in marginal bone change and patient-reported outcomes are susceptible to confounding by baseline periodontal status, implant positioning, prosthetic design, and follow-up duration—factors that were variably reported across studies. For clinicians, these findings support a context-driven approach to abutment selection (esthetic single-tooth goals favor TiB; structural and angulation needs in full-arch cases favor MU), and they underscore the need for standardized outcome definitions, consistent periodontal baseline reporting, validated PROMs, and adequately powered head-to-head randomized trials to establish causal comparisons.

## Conclusion

Both TiB and MU abutments are effective in supporting immediate-loading protocols when primary stability is achieved. In the currently available literature, TiB abutments are associated with lower mechanical maintenance and superior esthetic ratings in single-tooth/short-span restorations, while MU abutments remain the pragmatic choice for multi-implant and full-arch rehabilitations. Observed differences in marginal bone changes and patient-reported outcomes are small and likely influenced by case selection and reporting heterogeneity. Primary implant stability and careful prosthetic planning are critical determinants of immediate-loading success regardless of abutment choice. Well-designed, standardized head-to-head randomized trials with consistent outcome measures and adequate follow-up are required to confirm these findings and guide definitive clinical recommendations.

## Data Availability

The data supporting the results of this study can be obtained from the corresponding author upon reasonable request.
